# IDO-GCN2 and autophagy in inflammation

**DOI:** 10.18632/oncotarget.4846

**Published:** 2015-07-11

**Authors:** Tracy L. McGaha

**Affiliations:** Department of Medicine, Medical College of Georgia, Augusta, GA, USA

**Keywords:** Immunology Section, Immunity, Immune response

Inflammation is a disruptive force in the tissue microenvironment driving extracellular matrix reorganization, production of noxious effector molecules (superoxide, nitric oxide, peroxynitrite, ect.), hypoxia, and active consumption of nutrients required for biosynthesis including amino acids. Indoleamine 2,3 dioxygenase 1 (IDO1) is an intracellular, interferon inducible, enzyme that catalyzes oxidative metabolism of compounds containing indole-rings, in particular the essential amino acid tryptophan [1]. IDO1 was identified as a key driver of acquired immunologic tolerance in the periphery and has been extensively studied for its ability to suppress T cell responses in the context of tumors and cancer-driven T cell dysfunction [2]. However, in 2012 we reported that IDO1 was induced in response to apoptotic cell phagocytosis in tissue-resident macrophages [3]. In this context it was clear the major effect of IDO1 was to limit macrophage inflammatory potential after apoptotic celldriven activation, thereby preventing inflammatory autoimmunity. This led to an interest of my laboratory in the mechanistic role of IDO1 in pathology associated with systemic autoimmunity.

In the June 15^th^ issue of the Journal of Immunology we reported that interferon (IFN)γ-induced IDO1 is a critical factor limiting antibody-driven nephritis in mice modeling lupus nephritis and Goodpasture's disease [4]. In this pathology model, low doses of injurious antibodies drive a mild, self-limiting disease. However, if mice lacked IDO1 activity, this mild disease phenotype was converted to severe end stage renal disease with complete mortality. This remarkable finding illustrates the key role IDO1 plays as a feedback mechanism preventing excessive pathology in immune tissue injury. IDO1 is known to regulate inflammation by the physical act of tryptophan consumption inducing the evolutionarily conserved kinase general control nonderepressible 2 (GCN2). Accordingly, GCN2KO mice completely phenocopied IDO1KO mice with a significant enhancement of sensitivity to antibody driven renal injury. Surprisingly, early IDO1 expression in the kidney was not in the infiltrate (i.e. macrophages and neutrophils), but was rather in the specialized stromal epithelial cells that form the filtration barrier in the renal glomerulus (podocytes). Moreover, we found that the apparent function of IDO1 and GCN2 in this context was to protect the podocytes from apoptotic cell death, although this signaling circuit also had a role in reducing the numbers of infiltrating innate immune effector cells and induction of inflammatory cytokines [4].

GCN2 is an amino acid sensor that binds uncharged tRNAs that accumulate when intracellular amino acids are depleted, resulting in auto-phosphorylation and activation of kinase activity that targets translation elongation machinery modulating protein production. The functional relationship of GCN2 and inflammation is poorly understood. However it has been recently suggested that GCN2 is a potent driver of autophagy [5]. Correspondingly, we found that antibody injury induced an IDO1 and GCN2-dependent autophagy response in podocytes; moreover, if we inhibited the autophagy pathway *in vitro* podocytes were more sensitive to oxygen radical-driven cell death and a temporary inhibition of autophagy led to significant pathology *in vivo* after antibody injury [4]. Importantly, if we treated mice with agonists of either IDO1 or GCN2 activity autophagy was induced and animals were protected from otherwise fatal renal inflammation. The fact that we saw similar IDO1 and stress gene induction in biopsies obtained from patients suffering from a range of inflammatory kidney diseases suggests that IDO1-GCN2-autophagy signals may be a common circuit induced in human inflammatory disease which could be potentially targeted for therapeutic benefit.

Tumor cells often express IDO1, which is thought to potently suppress cytotoxic T cell responses and inflammatory dendritic cell maturation in situ generating a checkpoint that inhibits effective anti-cancer immunity [1]. Likewise, tumors adapt to low amino acid and glucose availability in the microenvironment by a stress signaling mechanism dependent on GCN2 [6]. In the tumor environment amino acid depletion can occur by multiple mechanisms including enzymatic degradation and metabolic demands of uncontrolled replication that outstrip the supply. Thus, unlike acute inflammatory environments, in tumors the requirement for adaptation to limited nutrient availability is constant and tumor cells that adapt most readily to these demands will have a competitive advantage. Moreover, it is clear that autophagy is a strategy that many tumors employ to deal with environmental stress [7]. Thus, targeting mechanisms that drive autophagy would be attractive for a variety of cancers.

An agonist of GCN2 has been identified (halofuginone hydrobromide) which we have found drives potent suppression of lupus, likely due to activation of GCN2 (unpublished observations). Likewise, identification of pharmacologic antagonists of GCN2 as immune activators is an area of intense interest and it is likely the future will yield an array of GCN2 blockers. Unexpectedly, the side effects of manipulating such a fundamental sensing system appears to be modest as GCN2KO mice are viable and show no overt phenotype, and treatment with halofuginone is associated with minor complications in the clinic. However care must be taken, as the long-term effects of GCN2 signal manipulation are unknown. Nevertheless, the accumulating body of evidence suggests IDO1 and GCN2 activity are attractive targets and we will likely see IDO-GCN2 targeted therapy emerge as an important tool in inflammatory disease in the future.
